# The Chiral 1:2 Adduct (*S*)_S_(*S*)_C_(−)_589_-Ethyl 2-Phenylbutyl Sulphide-Mercury (II) Chloride:(−)_589_[(*S*)_S_(*S*)_C_-Et(2-PhBu)S.(HgCl_2_)_2_]. Stereoselective Synthesis, Asymmetric Oxidation, Crystal and Molecular Structure and Circular Dichroism Spectra

**DOI:** 10.3390/molecules25153398

**Published:** 2020-07-27

**Authors:** Paolo Biscarini, Ivano Bilotti, Francesco Ferranti, Alessia Bacchi, Giancarlo Pelizzi, Marian Mikołajczyk, Jozef Drabowicz

**Affiliations:** 1 Department of Industrial Chemistry CHIMIND, Alma Mater Studiorum Bologna University, V.le Risorgimento 4, 40136 Bologna, Italy; ibilotti@gmail.com (I.B.); drabow@gmail.com (F.F.); 2 Dipartimento di Chimica Generale ed Inorganica, Università di Parma, V.le delle Scienze, 43100 Parma, Italy; chimic6@ipruniv.cce.unipr.it; 3 Center of Molecular and Macromolecular Studies, Polish Academy of Sciences, Division of Organic Chemistry 90-363 Lodz, 112 Sienkiewicza, Poland; marmikol@cbmm.lodz.pl; 4 Jan Dlugosz University in Czestochowa, Armii Krajowej 13/15, 42-200 Czestochowa, Poland

**Keywords:** sulphide mercury chloride complex, chirality, X-ray crystallography, asymmetric oxidation, circular dichroism, absolute configuration

## Abstract

Optically active (−)_589_ethyl (*S*)-2-phenylbutyl thioether, (−)(*S*)_C_-Et(PhBu)S (**I**), and its new diastereoisomeric mercury (II) chloride adduct, 1:2, (−)[(*S*)_S_(*S*)_C_-Et(PhBu)S.(HgCl_2_)_2_]_2_, (**II**) were stereoselectively synthesized; the absorbance (UV) and circular dichroism (CD) spectra were measured and the crystal and molecular structure of complex (**II**) was determined by single-crystal X-ray diffraction. Two different Hg centres are present whose coordination environments are built by two short bonds to chloride ligands in one case, and to one chloride and one sulphur in the other one. These originate digonal units. Electroneutrality is achieved by a further chlorine, which can be considered prevalently ionic and bonded to the two Hg centres, forming square bridging systems nearly perpendicular to the digonal molecules. The coordination polyhedra can be interpreted as 2 + 4 tetragonally-compressed octahedra with the four longer contacts lying in the equatorial plane. IR spectroscopic data are consistent with the presence of one bent and one linear Cl–Hg–Cl moiety. The absolute configurations at both stereogenic centres of the formed diastereoisomeric complex (**II**) are (*S*). The (*S*)_S_ absolute configuration at the stereogenic sulphur atom bonded to the mercury(II) atom in complex (**II**) has been related with the negative Cotton effect assigned in its circular dichroism (CD) spectrum to a charge-transfer transition at ca. 230 nm. The stereoselective oxidation of (***I***) and (**II**) with hydrogen peroxide, induced by the stereogenic carbon atom (*S*)_C_ of the enantiopure sulphide, gave (−)_598_ethyl (*S*)_C_-2-phenylbutyl(*S*)_S_-sulphoxide, (−)_598_[(*S*)_S_(*S*)_C_-Et(PhBu)SO], (**III**), having 18.1% de. Oxidations carried out in the presence of a 200 molar excess of mercury(II) chloride gave (−)_598_ethyl (*S*)_C_-2-phenylbutyl(*R*)_S_-sulphoxide, (−) _598_[(*R*)_S_(*S*)_C_-Et(PhBu)SO], (***IV***) with 31% de, showing the cooperative influence of mercury(II) chloride on the selectivity of the oxidation reaction.

## 1. Introduction

Mercury(II) halide complexes of chiral sulphides have been studied in the past to clarify their reactivity and their spectroscopic, structural and stereochemical properties [1,2,3,4,5,6]. It is known that sulphide or sulphoxide–mercury(II) chloride adducts can be formed with different L/M molar ratios, 1/1, 1/2 or 2/3. It is also well known that the nature of alkyl- or aryl chains of the sulphides has a substantial influence on the stability and molar ratio, L/M, of these complexes.

It is reasonable to assume that the observed composition of the complexes depends on the conformation, steric hindrance and/or electronic effects of the chains bonded to the sulfenyl sulphur atom. It is also reasonably to expect that complexes having different stoichiometry and stereochemistry should have different stability. On the other hand, due to the dissociation of the complexes formed in a solution, some equilibria exist between the complexes, mercury(II) chloride and a sulphide or a formed sulphoxide [7]. It was observed that the 1:1 sulphide-mercury (II) chloride dimeric adducts show molecular and crystal structures strongly different from the 1:2 polymeric adducts [4]. These complexes, in the solid state, have a pseudo- tetrahedral geometry around the sulphur atoms and various coordinations at the mercury atoms with bridging chlorine atoms bonding the metals in chains or layers [2,5]. The addition of mercury(II) chloride to optically active (−)(*S*)-methyl(2-phenyl) butyl sulphide, (−)(*S*)-Me(Ph-Bu)S, gave the diastereoisomerically pure 1:1 complex [(−)(*S*)_S_(*S*)_C_-Me(PhBu)S∙HgCl_2_]_2_. This complex constitutes the first example of a chiral tricoordinated sulphur compounds in which a stereogenic sulphur atom having the (*S*)_S_ absolute configuration was generated by the formation of the sulphur-metal bond [6]. This experiment showed for the first time that chirality could be predetermined and transferred from a stereogenic carbon atom to a prochiral sulphur atom of an asymmetrical sulphide via the addition of the suitably selected metal. In this context, it is interesting to note that fractional crystallization of mercury(II)chloride complexes of optically active sulphoxides of low enantiomeric excesses improves these values due to spontaneous enrichment during crystallization [8].

Taking into accounts these facts, we decided to check how other optically active sulphides, *R_1_R_2_S, will behave in analogous reactions of the formation of the complexes with HgCl_2_. We wanted to determine their molecular structure and circular dichroism spectra, with hope that such determinations will show the influence of an alkyl group of the thioether on the stereochemistry and the molar ratio, L/Hg, in the formed diastereoisomeric complexes.

Therefore, in this contribution we would like to report the results of our studies on the formation of complex (**II**) and on the stereochemical outcome of its oxidation to the diastereomeric sulphoxide, *R_1_R _2_* SO, (**III**) and (**IV**). By the studies on the complex formation, we wanted to establish the influence of the absolute configuration of the stereogenic carbon atom and of the chain conformation of the sulphide on the stereoselectivity of the addition of mercury(II) chloride to the prochiral divalent sulphur atom. In this way, we wish to foresee the absolute configuration of the newly created stereogenic centre at the three-coordinated sulphur atom of complex (**II**). On the other hand, experiments on the oxidative conversion of complex (**II**) were considered as a possibility of the observation of the so-called double asymmetric induction. This should occur with the simultaneous transfer of chirality from the carbon atom in (−)(*S*)_C_-Et(PhBu)S, (**I**) and from the new stereogenic sulphur-mercury bonded group, in the diastereoisomeric mercury(II)chloride adduct, 1:2, *R_1_R_2_*S.(HgCl_2_)_2_, (**II**) to the newly created stereogenic sulfinyl sulphur atom in the optically active sulphoxide *R_1_R_2_*SO, (**IV**) is taken into consideration. It was also interesting to see if the transfer of chirality is accompanied by retention or inversion of configuration at the sulphur stereogenic centre of the *R_1_R_2_*S-(Hg Cl_2_)_2_ complex (**II**), and of the sulphoxide ^*^R_1_R_2_*SO, (**IV**).

## 2. Experimental

Optical rotations were measured on an NPL Bendix (Bendix Electronics Ltd., Nottingham England) automatic polarimeter. The UV spectra in various solvents were recorded on a Jasco UVIDEC 650 spectrophotometer (Jasco, Tsukuba, Japan). The CD spectra were measured with a Jasco 500 A and J-810 spectropolarimeter (Jasco, Tsukuba, Japan). The solvent and commercial reagents were dried and distilled by conventional methods before use. The diffuse reflectance circular dichroism spectra, DRCD, were obtained for the solid complexes in order to relate the absolute configuration, determined by X-ray, to the observed Cotton effects [9]. The ^1^H NMR and ^13^C NMR spectra were taken using a 200 MHz Varian Gemini (Varian, Palo Alto, CA, USA and a 300 MHz CXP Bruker spectrometer (Bruker Gmbhr, Reinstetten, Germany) The chemical shifts refer to TMS as an internal standard and are given in p.p.m. IR spectra were recorded with a Perkin Elmer 225 spectrophotometer (Perkin Elmer, Wien, Austria) between 4000 and 200 cm^−1^ in nujol and hexachlorobuta-1,3-diene mulls. Thin polyethylene films were placed between the mull of the complex and the CsI windows to avoid halogen exchange.

### 2.1. Preparation of the Compounds

(*S*)_C_ (−)-Ethy-l,2-phenylbutyl sulphide, (−)(*S*)_C_-Et(PhBu)S, (*I*), b.p.83-85/0.7 mmHg, [α]^24^_D_ = +6.2 (C = 1.1%, ethanol 95%), α_D_ = +6.55 (neat, l = 1) was obtained as described [10]. The CD spectrum in ethanol: 261 nm, Δε = +0.11; 230 nm, Δε = −3.0; 212 nm, Δε = +3.7.

(*S*)_S_(*S*)_C_ (−)_589_ Ethyl 2-phenylbutyl sulphide-mercury(II) chloride (1/2), (−)[(*S*)_S_(*S*)_C_-Et(PhBu)S.(HgCl_2_)]_2,_ (*II*), was prepared by adding an ethanolic solution of the sulphide (***I***), [0.5 g, (2.6 mmol)], to 50 cm^3^ of an ethanolic solution of mercury(II) chloride, [1.6 g, (6 mmol)]. This gave a white precipitate, which was isolated by filtration and then slowly crystallized from ethanol (95%): The isolated white solid, yield 70%, had, m.p. 120 [α ]^24^_D_ = −2.4 (C = 1.0 %, ethanol 95%), 1.4 g,. *Anal.* Found: C, 19.45; H, 2.45; Hg, 54.0; S, 4.35%. Calc for C_12_ H_18_ Cl_4_Hg_2_ S: C, 19.55; H, 2.46; Hg, 54.4; S, 4.34%. The IR spectrum shows strong bands at. 348 cm^−1^ and 358 cm^−1^ assigned to the asym (Cl-Hg-Cl) two different SHgCl_2_ and HgCl_2_ groups. The UV and CD spectra of (**II**) in diluted solutions are similar to those of sulphide (***I***). In concentrated solution the CD spectrum shows a strong negative band at about 230 nm.

The DRCD spectra of the solid sample (**II**) show a negative Cotton effect at ca. 260 nm.

### 2.2. X-ray Analysis of (**II**)

X-ray diffraction analysis was performed at room temperature on a colourless crystal with a computer-controlled Siemens AED diffractometer (Siemens, Karlsruhe, Germany), at the very beginning of this research project; this is the reason why data processing and refinement are carried out with the software available at that time. Cu-Kα radiation was used in order to enhance anomalous dispersion effects enabling one to attribute the correct absolute configuration. The automatic peak search, centring and indexing procedures established an orthorhombic primitive lattice, and systematic extinctions unambiguously identified the space group as *P*2_1_2_1_2. No crystal decay was observed. The crystal data and details of data collection and refinement are reported in Table 1. The intensity data were processed by a peak-profile procedure and corrected for Lorentz and polarization effects. The phase problem was solved by using the Patterson superposition procedure of Shelxs96 [11] which allowed for locating the two metals, the sulphur and three chlorine atoms. The entire structure was retrieved by a few cycles of full-matrix least squares refinement and electron density map inspection using Shelxl96. Hydrogen atoms were added at calculated positions, riding on their carrier atoms. Several cycles of full-matrix least squares were performed on all reflections, using F^2^. After the last isotropic refinement, the empirical absorption correction of Walker and Stuart was applied [12]. Anisotropic thermal displacement parameters were refined for all non-hydrogen atoms. The correctness of the absolute configuration was tested by refining the Flack parameter, according to Shelxl96 protocol. Neutral atomic scattering factors were employed, those for non-hydrogen atoms being corrected for anomalous dispersion. The final geometry was analysed by the program PARST95 and the drawings were made with ZORTEP [13,14]. In addition to referring to the original literature, the data for comparison with other compounds were retrieved and analysed by the software packages of the Cambridge Structural Database [15,16]. The final fractional atomic coordinates for non-hydrogen atoms are given in Table S2 (see Supplementary Materials).

### 2.3. Oxidation Reaction

(−)(*S*)_S_(*S*)_C_-Ethyl-2-phenylbutyl sulphoxide, (−)(***S***)**_S_**(***S***)**_C_**-Et(PhBu)SO, (**III**), was obtained by oxidation of (***I***) by hydrogen peroxide with sulphuric acid as a catalyst in the absence or presence of few amounts of mercury(II)chloride.

(−)(*R*)_S_(*S*)_C_-Ethyl-2-phenylbutyl sulphoxide, (−)(*R*)_S_(*S*)_C_-Et(PhBu)**SO**, (**IV**), was obtained by oxidation of (***I***) in presence of a high amount of mercury(II) chloride.

*Oxidation of sulphide* (*I*)*:* 0.058 g of sulphide (***I***) [(0.3 mmol; [α]_589_ = −3.0 (acetone)] was dissolved in ethanol (3 mL, 98%). To this solution 50 mg of hydrogen peroxide (30%) and one drop of sulphuric acid were added at room temperature. The reaction mixture was kept at room temperature for additional 30 min. Next, the reaction mixture was diluted with water (50 mL) and the water phase was extracted with chloroform (4 × 15 mL). The combined chloroform extracts were washed with 5% aqueous potassium carbonate and water and dried over anhydrous magnesium sulphate. Evaporation of the solvent gave virtually pure sulphoxide (−)(*S*)_S_(*S*)_C_-Et(PhBu)SO, (**III**) (53 mg, 93%) having optical rotation [α]_589_ = −34.0 (c = 1.22, acetone). Analysis of the ^1^Hnmr absorption pattern of the methyl protons of the ethyl group bonded to the chiral sulfinyl sulphur atom (two triplets with δ_1_ = 1,2544 and δ_2_ = 1.3054) showed d.e. of 18.2%. The CD spectra of the sulphoxide (**III**) in n-heptane show a weak positive Cotton effect with a vibronic structure at ca. 260 nm, lowered under the zero line by a new negative one scarcely resolved Cotton effect at ca. 240 nm. This is followed by a strong negative one at 216.7 nm, Δε = −0.3. These Cotton effects distinguish sulphoxide (**III**) from sulphide (***I***) and its complex (**II**), and from sulphoxide (**IV**) obtained by oxidation of sulphide (***I***) in the presence of an excess of HgCl_2_ (see Figure 5).

Oxidation of the complex (II): 0.221 g of the complex (**II**) (0.3 mmol) was oxidized as described for the sulphide (***I***). From the solution, treated to eliminate mercury (II) chloride as described below, the sulphoxide (**III**) was isolated. It had [α]_589_ = −34.0 (c = 1.30 acetone) and de = 18.0%.

Oxidation of the sulphide (I) in the presence of 10 molar excess of mercury (II) chloride: The sulphide (***I***) [(0.194 g, 1 mmol; [α]_589_ = −3.0 (acetone)] and mercury (II) chloride (2.71 g, 10 mmol) were dissolved in ethanol (32 mL, 95%). To this solution hydrogen peroxide (150 mg, as 30% solution) and sulphuric acid (50 mg) were added at room temperature. Then the reaction mixture was kept at room temperature for 20 h. After removal of the solvent by evaporation at reduced pressure, the residue was dissolved in acetone. Addition of solid potassium carbonate to the acetone solution decomposed the residual complex of the formed sulphoxide and mercury(II) chloride. The removal of the formed solid, yellow, mercury oxide by filtration and evaporation of the solvent gave a crude sulphoxide (150 mg) having optical rotation [α]_589_ = −24.42. The crude product was purified by column chromatography on silica gel (15 g). Elution with petroleum ether (70 mL) followed by methylene chloride (50 mL) gave the starting sulphide (42 mg, [α]_589_ = −3.0). Elution with acetone (100 mL) gave the pure sulphoxide (**III**) (0.09 g, yield 42%). This sample had [α]_589_ = –33.5 (c = 1.36 acetone) and de = 18.4%.

Oxidation of the sulphide (I) in the presence of 200 molar excess of mercury (II) chloride: The sulphide (I) [(0.0389 g, 0.2 mmol); [α]_589_ = −3.0 (acetone)] and mercury (II)chloride (10.84 g, 40 mmol) were dissolved in ethanol (300 mL, 95%). To this solution, hydrogen peroxide (30 mg as the 30% solution) sulphuric acid (15 mg, 96%) were added at room temperature. The progress of the reaction carried out at room temperature was followed by TLC analysis. When the reaction was over, ethanol was evaporated and 500 mL of acetone was added to the residue. Solid potassium carbonate (25 g) was added to the formed solution and a heterogeneous mixture was shaken for 20 min. The solid part was filtered off and the acetone solution was concentrated by evaporation of the solvent. Ethyl ether was added to the residue (~200 mg) and the precipitated solid was filtered off. Evaporation of the ether solution gave a liquid which was finally purified on a preparative TLC plate (petroleum ether-methylene chloride(1:1) as a developing solvent system) giving 21.6 mg (50%) of the pure sulphoxide (−)(*R*)_S_(*S*)_C_-Et(PhBu)SO, (***IV***), having [α]_589_ = –50.0 (acetone). Analysis of the ^1^Hnmr absorption pattern of the methyl protons of the ethyl group bonded to the chiral sulfinyl sulphur atom (two triplets with δ_1_ = 1.2382 and δ_2_ = 1.2900) showed de = 31.4%

The CD spectra of the sulphoxide (−)(*R*)_S_(*S*)_C_-Et(PhBu)SO, (**IV**) in n-heptane show a weak Cotton effect with vibronic structure at *ca* 260nm, Δε = +0.25 nm, and a new positive one at 233 nm, Δε = +0.30, which distinguishes the sulphoxide (**IV**) from sulphoxide (**III**), sulphide (***I***) and its complex (**II**). These Cotton effects were followed by a negative one at 215 nm, Δε = −6.5 and two positive ones at 205 and 198 nm, Δε = +2.2 and Δε = +6.0, respectively.

## 3. Results and Discussion

### 3.1. Formation Reaction

Upon addition of the chiral non-symmetric sulphide (**I**) to a concentrated solution of mercury(II) chloride in ethyl alcohol at room temperature, the new diastereoisomeric complex with mercury(II) chloride, (−)[(*S*)_S_(*S*)_C_-Et(PhBu)S.(HgCl_2_)_2_]_2_, (**II**), was instantaneously formed. The molar optical activity of the isolated complex in ethanol was [α]_D_ = –2.4. This value grows upon addition to the solution of an excess of mercury chloride, thus showing the progressive formation of a new stereogenic centre in the molecule.

**Scheme 1:**
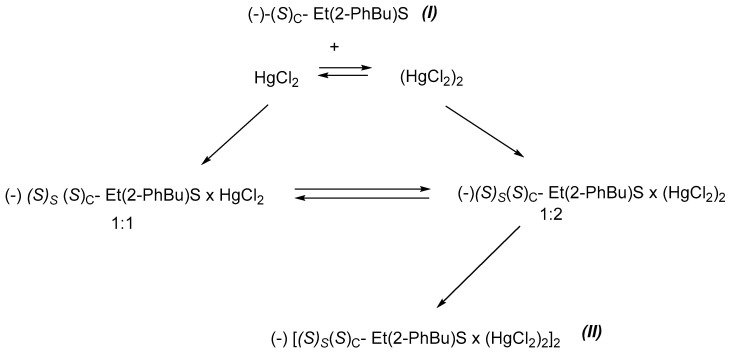
An equilibrium between the free ligand (***I***) and HgCl_2_.

**Scheme 2:**
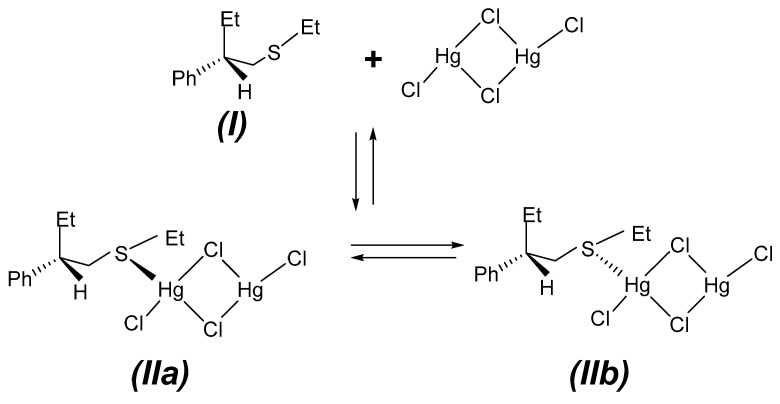
Diastereoisomers of sulphide (**I**) with mercury chloride.

It can, therefore, be supposed that in a solution complex (**II**) is in an equilibrium with the free ligand (***I***) and HgCl_2_, as shown in Scheme 1. The solid state IR spectra, analytical results and X-ray diffractometric characterisation of the complex indicated that its stoichiometry was 1:2. The formation of the complex can reasonably be explained if one assumes that in a concentrated solution mercury (II) chloride exists in the dimeric form and adding it to one of the diastereotopic faces of the prochiral sulphur atom of the optically active sulphide (**I**) gives an intermediate complex, which crystallises in polymeric 1:2 form:

The addition of mercury(II) chloride to the optically active sulphide (**I**) may yield two diastereoisomers, (*S*)_S_(*S*)_C_ -, (**II*a***), and (*R*)_S_(*S*)_C_-Et(PhBu)S∙(HgCl_2_)_2_, (**IIb**), where *R* or *S* refers to the absolute configuration of the sulphur and carbon atom, C(3), of the sulphide. The metal could bind to the prochiral sulphur atom from two different directions and create the two diastereoisomers, as shown in Scheme 2, crystallizing by an asymmetric second order process as (*S*)_S_(*S*)_C_-Et(PhBu)S.(HgCl_2_)_2_, (**II*a***), examined by X-ray.

### 3.2. Infrared Spectra of the Solid Complex

It has been shown that in the infrared spectra of 1:2 adducts of mercury(II) chloride with a variety of sulphides two strong absorption bands between 300 and 350 cm^−1^ occur whereas the spectrum of the 1:1 complex shows only one absorption band at about 350 cm^−1^ [3,6]. The infrared spectrum of the complex (**II**) recorded in nujol mulls between 1300 and 200 cm^−1^ shows the strongest band at 348 cm^−1^ due to γ_asym_ (Cl–Hg–Cl) of the SHgCl_2_ group, with a shoulder at lower energy and a medium- intensity band at 358 cm^−1^, assigned to the γ_asym_ (Cl–Hg–Cl) of the mercury(**II**) chloride groups with a different effective coordination. This spectrum, which is very similar to the IR spectra of the analogous 1:2 adducts of dialkyl sulphides with mercury(II) chloride, such as Et_2_S∙2HgCl_2_, clearly indicates the 1:2 ratio in the complex (**II**) [10]. It should be noted here that the analysis of the frequency values indicate the presence of one bent and one near linear Cl–Hg–Cl unit. As we will see below (see X-ray analysis) these frequencies correspond to the Cl–Hg–Cl angles of 100.2 and 173.2^°^.

### 3.3. Molecular and Crystal Structure by X-ray Diffractometry

**Figure 1:**
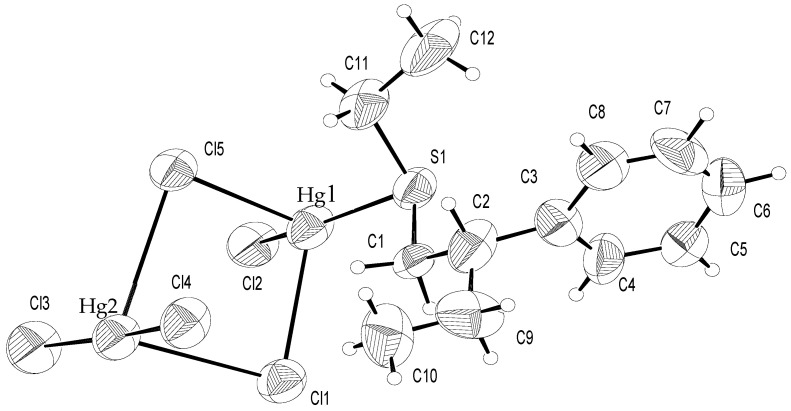
Asymmetric unit and labelling of the crystal structure of compound (**II**). Thermal ellipsoids are at the 50% probability level.

The adduct (Et(EtCHPhCH_2_)S)Hg.(HgCl_2_)Cl is polymeric in the crystal, and is constituted by SHgCl^+^∙HgCl_2_ units, assembled with the assistance of Cl^−^ anions. Figure 1 shows the contents of the asymmetric unit along with the atom labelling, and Table S2 (see SI) lists bond distances and angles. Both metals are surrounded by six donors in a highly distorted octahedral arrangement very similar to the one found in Et2S∙2HgCl_2_ [2].

While only Hg1 is bonded to the sulphur ligand, for both Hg1 and Hg2 the coordination environment is characterised by two short bonds, to Cl2 (2.366(7)Å) and S (2.397(7)Å) for Hg1, and to Cl3 (2.276(8)Å) and Cl4 (2.300(7)Å) for Hg2, which originate digonal units, more distorted for Hg1 (154.9(3)°), rather regular for Hg2 (173.2(4)°). The electroneutrality of the adduct is achieved by a further chlorine, which can be considered prevalently ionic [2]. In the crystal, this atom is placed in two independent positions on the two-fold axis, Cl1 and Cl5. The anions are bonded to Hg1 and Hg2, at distances falling in the range 2.767–3.146 Å, as observed for similar compounds forming square bridging systems nearly perpendicular to the digonal molecules, with internal angles ranging from 85.1(2)° to 96.9(2)° [6]. The tetraatomic ring Hg1–Cl1–Hg2–Cl5 is slightly folded (8.5°) along the Cl1–Cl5 vector. Since both anions lie on symmetry elements, two equivalent adjacent rings, reaching a distorted tetrahedral coordination, with angles Hg–Cl–Hg ranging from 80.3(3) to 145.9(2)° for Cl1 and from 77.8(2) to 141.3(2)° for Cl5 share each anion. Both metals complete hexa-coordination by means of long interactions ranging between 3.162 and 3.333 Å with atoms Cl2 and Cl4 belonging to symmetry related molecules. The coordination polyhedra can be interpreted as 2 + 4 tetragonally compressed octahedra with the four longer contacts lying in the equatorial plane. However, the real picture is more complex in both cases, with atoms deviating up to 0.45 Å and 0.39 Å from the least-squares average equatorial plane containing Hg1 and Hg2, respectively. It is convenient to describe the crystal polymeric packing on the basis of the interactions between digonal molecules Et(EtCHPhCH_2_)SHgCl^+^ and HgCl_2_, and of the role played by the different chlorines in these interactions.

**Figure 2:**
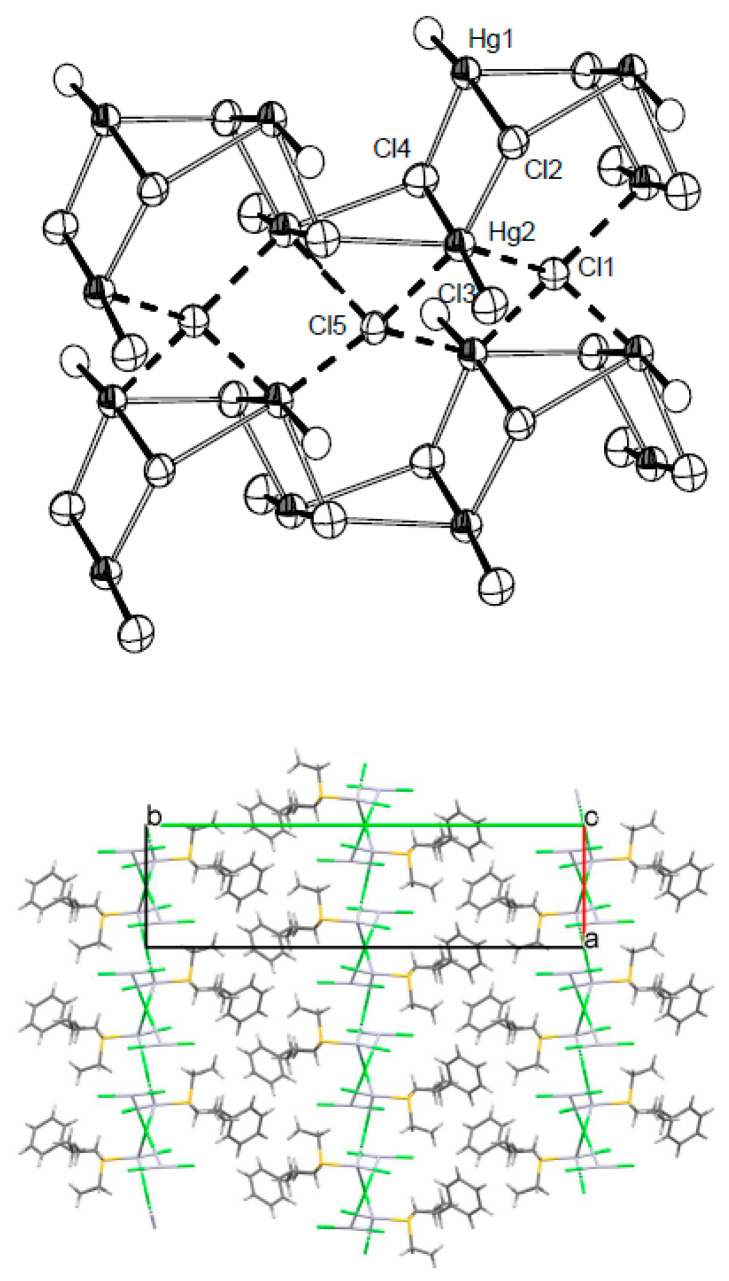
Top: Arrangement of the SHgCl^+^∙HgCl_2_ units (black bonds) in puckered ribbons (white bonds), assembled with the assistance of Cl1 and Cl5 anions (dashed bonds). Bottom: polymeric chains viewed along the c axis.

**Figure 3:**
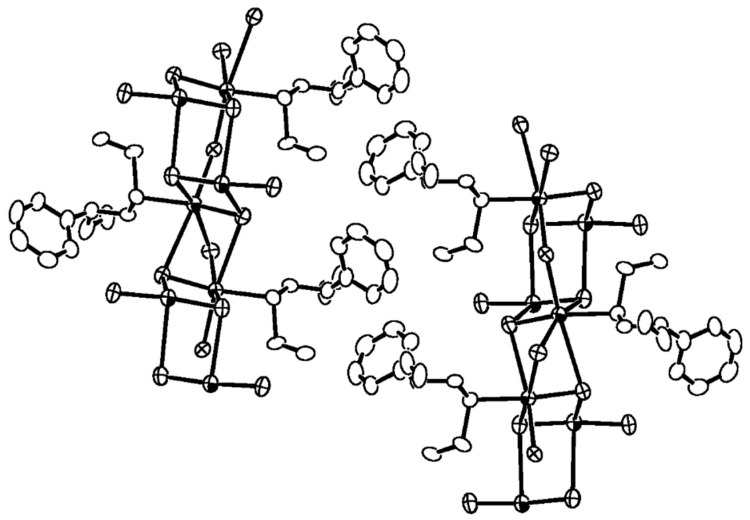
Packing arrangement of (**II**), showing interdigitation of organic moieties in the assembly of polymeric ribbons.

As shown in Figure 2, Cl2 and Cl4 are strongly bonded to Hg1 and Hg2, respectively (digonal molecules, indicated with thick bonds), and reach a trigonal coordination by bridging two other neighbours metals, namely Hg1 (−x, −y + 1, z) and Hg2 (x, y, z − 1) for Cl2 and Hg1 (x, y, z + 1) and Hg2 (−x + 1, −y + 1, z) for Cl4. This originates tetraatomic rings in which the metals occupy opposite vertices and in which the edges corresponding to the short bond are shared between adjacent rings. The polymeric structure can be viewed as the joining of Et(EtCHPhCH_2_)S HgCl^+^∙HgCl_2_ dimers in a head-to-head, tail-to-tail fashion. The resulting cationic ribbons are highly puckered since the interactions between dimers occur perpendicularly to the dimer plane. The anions Cl1 and Cl5 are intercalated in tetracoordinated sites and have the two-fold function of stabilising the puckering pattern by bridging pairs of metals which delimitate the convex sections of the ribbon, and of favouring the stacking of the ribbons in the *a* direction, forming inorganic layers in the *ac* plane. On the basis of their role in the crystal packing, it is likely that Cl1 and Cl5 act as templates in determining the organisation of the packing motif. The remaining non-ionic chlorine, Cl3, does not participate to any intermolecular contact, and it is topologically equivalent to the sulphur ligand in trimming the ribbon lateral edges. (Figure 3) Lipophilic interactions due to the aromatic part of the ligand occur between layers in the *b* direction.

The above motif is partially present also in the packing of the achiral Et2S∙2HgCl_2_ where short five-ring ribbons can be found organised in layers [2]. The main differences between the crystal arrangements of the two compounds involve the anionic chlorine, which in Et2S∙2HgCl_2_ is tricoordinated and loses part of its functionality of inter-ribbons stabiliser, and the HgCl_2_ linear molecule, which in Et2S∙2HgCl_2_ employs both its chlorines in the coordination of neighbouring metals.

Another interesting comparison can be made with the 1:1 adduct Me(EtCHPhCH_2_)S∙HgCl_2[_[6],^5^ containing a chiral diastereotopic thioether ligand very similar to the present one. The complex contains two chiral centres, the carbon C2, whose configuration is (S)c, and the coordinated sulphur, which can result either (*S*)_S_ or (*R*)_S_ depending on the stereochemistry of the coordination reaction. In both compounds the attack of the prochiral sulphur to the metal occurs in the direction which induces at the sulphur the same absolute configuration as the stereogenic carbon has. In the 1:1 adduct this was explained by the fact that the observed stereochemistry allowed to attain a suitable ligand conformation which stabilises the complex by means of π interaction, retained in the crystal structure, between the metal and the phenyl electronic cloud. The X-ray analysis shows that these interactions stabilize the (*S*)_S_(*S*)_C_ diastereoisomer in respect of the (*R*)_S_(*S*)_C_ one. The differential energy of the two possible diastereoisomers and stereochemistry of the intermolecular association interactions are the driving forces of the stereoselective synthesis and stabilization of the complex.

Assignment of the carbon absolute configuration was made on the basis of chemical arguments based on the synthesis of (*S*)(−)Et(PhBu)S, (***I***), starting from optically pure (S)(+)-2-phenylbutanoic acid [5]. Since subsequent replacement on the chiral centre occurred with retention of the initial configuration, this knowledge allows the (*S*)_C_ configuration to be assigned at C(3). At the same time, after crystallographic resolution, the sulphur chirality can be fixed accordingly as (*S*)_S_. In the present compound, **II*a***, the ligand adopts an extended conformation with a C11–S1–C1–C2 torsion angle of −69(2)°, compared with the value of −165(1)° found in the 1:1 adduct. The interaction with the phenyl ring is replaced by the intra-ribbon bond to Cl2 (−x, −y − 1, z), which is reinforced by the action of Cl1, bridging Hg1 and Hg1 (−x, −y − 1, z). This does not rule out the possibility that the aromatic ring is important in favouring the formation in solution of the (*S*)_S_(*S*)_C_ diastereoisomer, but suggests that the association between Et(EtCHPhCH_2_SHgCl^+^∙HgCl_2_ dimers might occur via an “in line” nucleophilic substitution of Cl2 on Hg1 (−x, −y + 1, z), and Cl2 (−x, −y + 1, z) on Hg1, assisted by the bridging action of Cl1.

### 3.4. Absorbance and Circular Dichroism Spectra

**Figure 4:**
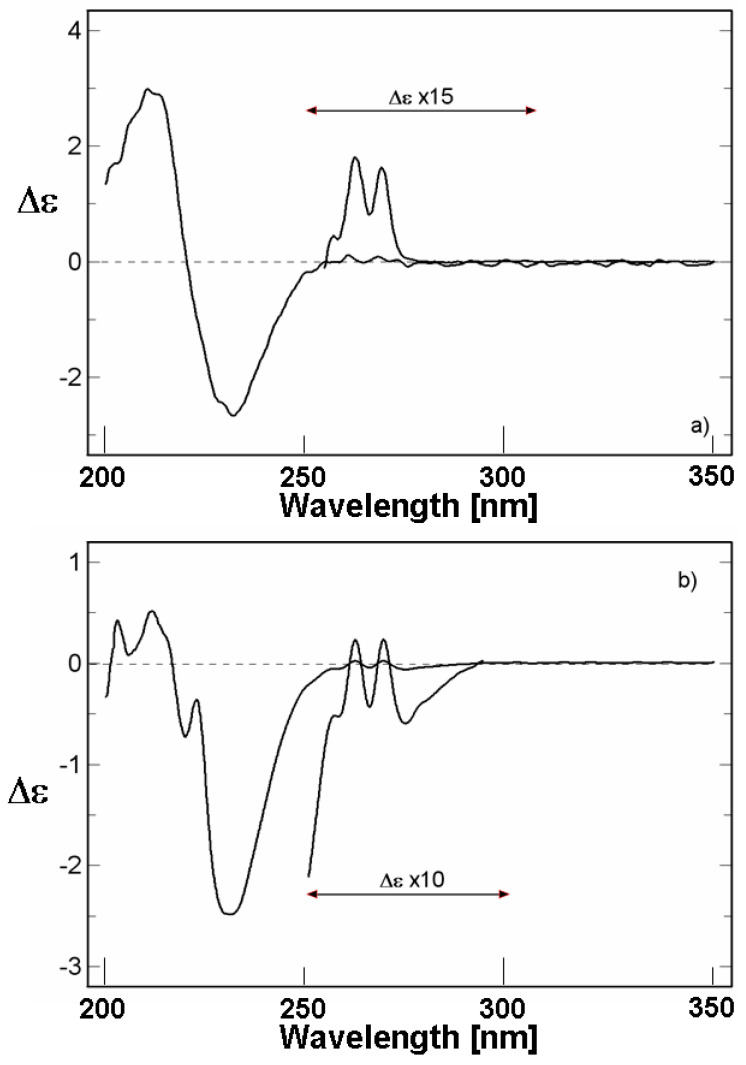
(**a**) CD spectrum of a diluted ethanolic solution of (−)[(*S*)_S_(*S*)_C_-Et(PhBu)S.2HgCl_2_,(**II**); (**b**) CD spectrum of an ethanolic solution of (−)[(*S*)_S_(*S*)_C_-Et(PhBu)S.2HgCl_2_,(**II**) after the addition of 200 mmol of HgCl_2_.

The formation of the chiral diastereopure complex (**II**) according to Scheme 1 is supported by the CD spectrum. The absorbance spectra of monosubstituted benzene derivatives show a weak first vibrationally structured band at ca. 260 nm assigned to the B_2u_ benzene-like transition. Additionally, less structured bands are observed at higher energies. The CD spectrum closely corresponds to the absorbance spectra, showing more clearly the energies and intensities of the expected transitions [10]. Three bands and one inflection corresponding to the four electronic transitions can be detected also in the CD spectrum of the sulphide-mercury(II) chloride complex (**II**). In diluted solutions, this spectrum as given in Figure 4a is very similar to that of the sulphide (**I**) [17]. It shows a positive Cotton effect with a vibronic structure, assigned to the B_2u_ transition of the phenyl group of the ligand at about 260 nm, a negative band at 232 nm related with the A_1_ and A_2_ electrically forbidden transition of the sulphide chromophore, a positive one at 210 nm related to the B_1u_ benzene-like transition, and an inflection at about 205 nm related to the allowed sulphide transition.

As for the sulphide (**I**), the (*S*)_C_ absolute configuration can be correlated with the sign of these three bands. In a diluted solution, complex (**II**) seems to be strongly dissociated and it is not possible to establish the Cotton effect due to the formation of the S–Hg bond. However, as it is shown in Figure 4b, by the addition of an excess of mercury(II) chloride the Cotton effect at 260 nm progressively passes into the negative part of the spectrum, being lowered by another band which grows at 230 nm as a strong negative Cotton effect, g = −2 × 10^−3^ for the ratio sulphide/HgCl_2_, 1/200. This new negative band can be tentatively assigned to a CT transition which grows upon addition of an excess of mercury(II) chloride, while, at the same time, the A_1_ and A_2_ CD band at 232 nm disappears; this is indicative of mercury(II) chloride addition to the sulphide and formation of a new S–Hg bond. These changes in the CD spectra also demonstrate the formation of a new stereogenic centre on the pseudo-tetrahedral sulphur atom bonding the metal.

A very similar Cotton effect related with a CT transition was observed earlier in the CD spectrum of the 1:1 complex of optically active (−)(*S*)_S_(*S*)_C_ methyl 2-phenylbutyl sulphide with mercury (II)chloride for which the (*S*) absolute configuration at the newly created chiral sulphur atom was established [6]. This similarity clearly indicates that the isolated diastereoisomer of the complex (**II**) also has the (*S*)_S_ absolute configuration at the sulphur atom as shown in the solid state by the molecular structure description.

The molecular structure of ethyl-2-phenylbutyl sulphide-mercury(II) chloride complex, 1/2, (**II**), shows that the (*S*)s absolute configuration at the sulphur atom has been obtained. In the CD spectra, the negative Cotton effect due to the CT of the 1:2 complex at ~230 nm is related with the (*S*)_S_ absolute configuration on a new stereogenic sulphur centre. This correlation demonstrates that the diastereoisomer (−)[(*S*)_S_(*S*)_C_-Et(PhBu)S∙2HgCl_2_]_2_, (**IIa**), (Scheme 2), has preferentially been formed and that a stereoselective addition of mercury to the prochiral sulphide sulphur atom has been achieved. The complex can be crystallized in the presence of a large excess of mercury chloride. In solutions near to saturation, the equilibria shown on Scheme 1 are shifted to the formation of the complex, which crystallizes following an asymmetric second order crystallization process. The same configuration is maintained in the solid state, as shown by molecular structure X-ray determination.

The crystal structure determination shows that the (*S*)_S_ configuration is always thermodynamically more stable and the stabilisation of the S–Hg bond is maintained in spite of the rotation of the aryl-substituted butyl chain, which thus establishes new interactions with the same groups of another parallel chain of the polymeric complex during crystal packing. The crystal packing energy is sufficiently high to overcome the phenyl-Hg interactions established during the formation process, and to determine the rotation of the phenyl-butyl chain around the C_1_-S bond, but not sufficient to break the S–Hg bond resulting from the first attack during the formation of the complex in solution.

### 3.5. Oxidation 

The oxidation of dialkyl-sulphides R^1^R^2^S with hydrogen peroxide and H_2_SO_4_ as a catalyst in presence of mercury(II)chloride are chemoselective giving complexes of mercury(II)chloride with pure sulphoxides as ligands without formation of the corresponding sulfones [3].

The oxidation of the chiral sulphide (**I**) with hydrogen peroxide and H_2_SO_4_ as a catalyst (Equation (1)) gives diastereoisomeric sulphoxide (−)(*S*)_S_(*S*)_C_ -Et(PhBu)SO, (**III**) having a de = 18.2%. This result shows that the oxidation of sulphide (**I**) is asymmetrically driven by the (*S*)_C_ absolute configuration of the carbon atom of the alkyl chain.

(1) $$\left(-)(S{)}_{C}\text{-}\mathrm{Et}(\mathrm{PhBu})S+H_{2}O_{2}\overset{\;H^{+}\;}{\to }\;(-)\;(S{)}_{S}(S{)}_{C}\text{-}\mathrm{Et}(\mathrm{PhBu})\mathrm{SO}\;\mathrm{de}18,\;2\%\;(I\right)$$

**Scheme 3:**
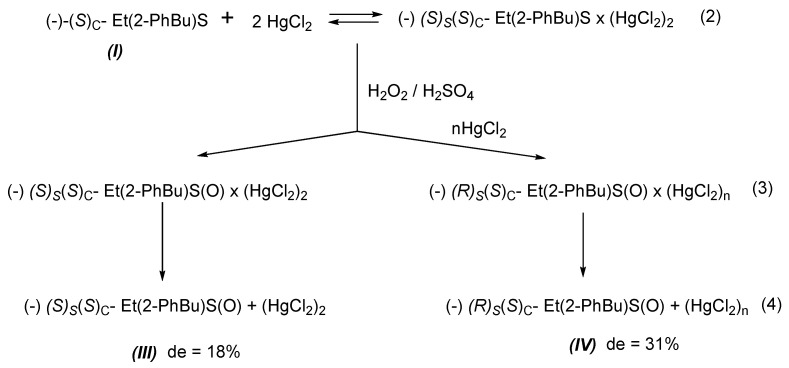
The oxidation of the 1:2 complex of (−)(R)_S_(S)_C_-Et(PhBu) with HgCl_2_ alone and in the presence of an excess of mercury (II) chloride.

The oxidation of the 1:2 complex alone and in the presence of an excess of mercury (II) chloride (Equations (2) and (3)) shows that a huge excess of mercury (II) chloride (1:200) induces a substantial increase in the diastereoselectivity of the reaction with inversion of configuration at a stereogenic sulphur atom. The oxidation of the 1:2 complex, without an excess of mercury (II) chloride gives sulphoxide (*S*)_S_(*S*)_C_, (**III**) with d.e = 18%, and with 200 molar excess of HgCl_2_, sulphoxide (*R*)_S_(*S*)_C,_ (**IV**) with d.e = 31%, respectively (Scheme 3).

The last oxidation shows that mercury chloride bonding sulphur atom with the (S)S absolute configuration cooperates significantly to the asymmetric synthesis of (−)(R)S(S)C-Et(PhBu)SO, (IV), driving the oxidation on the enantiotopic site of prochiral sulphur atom.

**Figure 5:**
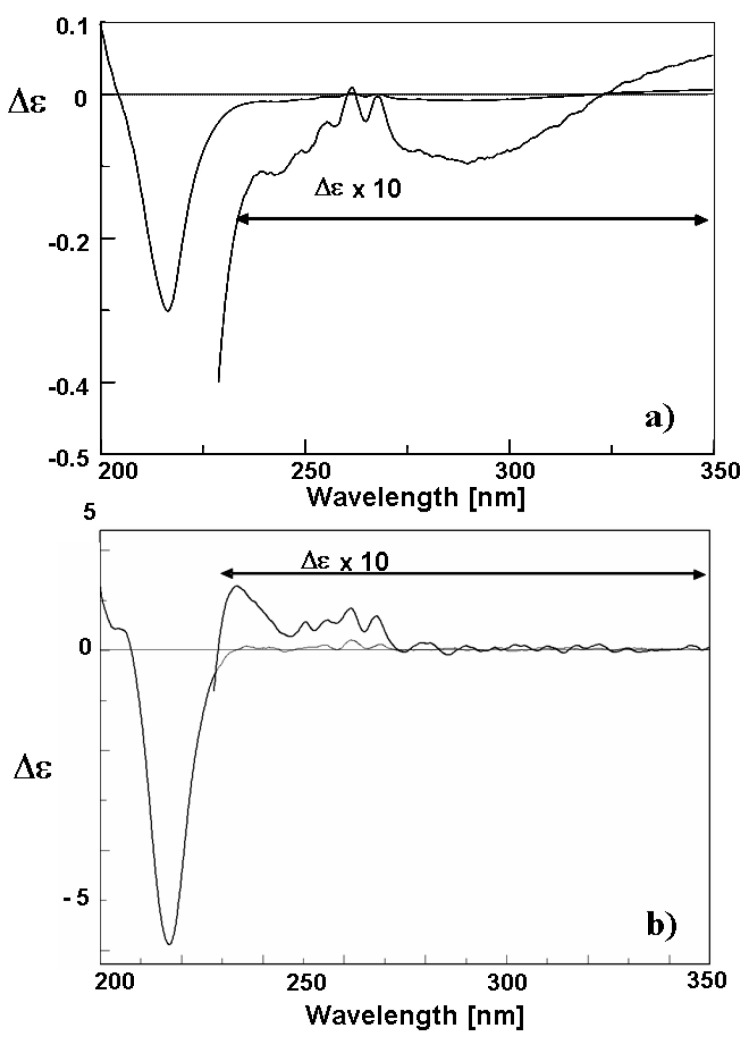
(**a**) CD spectrum of a n-heptane solution of: (−)(*S*)*_S_*(*S*)*_C_*-Et(PhBu)SO, (**III**) with de 18%; (**b**) CD spectrum of a n-heptane solution of: (−)(*R*)*_S_*(*S*)*_C_*-Et(PhBu)SO (**IV**) with de 31% obtained by decomposition of the complex of Reaction (3).

The (*R*)_S_ absolute configuration at the sulfinyl sulphur atom of this sulphoxide can be deduced from the CD spectrum (Figure 5) where a new positive Cotton effect at ~235 nm followed by another negative one at 220, a positive one at 205 and a strong positive one at ~198 nm are observed. When compared with the spectra of the corresponding sulphide, the CD spectra of sulphoxides show, a new positive or negative Cotton effect at ~235 nm. The presence of this new Cotton effect is commonly considered as an indicator of the absolute configuration at the sulphur atom, (*S*) _S_ if negative, (*R*)_S_ if positive [17]. In the examined case the configuration (*S*)_S_ in the complex of sulphide (**II**) becomes (*R*) _S_ in sulphoxides (**IV**), as shown by the positive sign of the Cotton effect at ca. 235 nm.

## 4. Conclusions

Chiral discrimination/recognition during the formation reaction of a chiral sulphur atom bonding a metal, and the crystallization of the resulting complex were investigated for mercury(II) chloride adducts with prochiral thioethers containing a stereogenic carbon atom. Unsymmetrical sulphide possess a prochiral sulphur atom. Therefore, they can form two enantiomers either by bonding of a metal atom in a complex or upon oxidation to the corresponding sulphoxide. It is obvious that the absolute configuration conformation and steric hindrance of substituents bonded to the sulphide sulphur atom should influence on the diastereoselectivity of the addition of mercury(II) chloride to a prochiral sulphur atom and the subsequent oxidation of the complex to a sulfoxide.

Enantiopure sulphides can form, as a result of bonding the metal atom to the diastereotopic site of the sulphur atom in a substrate, two diastereomers. In the oxidation of the formed complex only the enantiotopic lone electron pair should participate. This should generate a stereogenic oxidation sulfinyl sulphur atom by the cooperative assistance of mercury chloride in the diastereoselective reaction. Knowing the absolute configurations of the stereogenic atoms in the stable diastereomer of complex, it was possible to predict the prevailing site of the attack of an oxidant. X-Ray diffraction shows the absolute configurations of the complex (**II**) in the solid state. The circular dichroism was used to determine the absolute configurations of the compounds (**I**), (**II**), (**III**) and (**IV**) in a solution.

It was found that the transfer of chirality from the (*S*)_C_ carbon atom of a sulphide to the sulphur atom bonded to the mercury(II) chloride gives the (*S*)_S_ chirality at a newly created stereogenic centre on a mercury(II) complex. Therefore, the (*R*)_S_(*S*)_C_ sulfoxide is stereoselectively obtained by the direct oxidation of the (*S*)_C_ sulphide, whereas the oxidation with the cooperative help of 200 mmol of HgCl_2_ finally gives the sulfoxide having the (*R*)_S_ absolute configuration on the sulfinyl sulphur atom. Thus, the absolute configuration of the sulfinyl sulphur atom can be reversed by the prior generation of a complex between optically active sulphide and mercury(II) chloride.

## Figures and Tables

**Table 1:** Crystal data and structure refinement for (**II**).

Chemical Formula	C_12_ H_18_ C_l4_ Hg_2_ S
Formula weight	737.30
Temperature	293(2) K
Wavelength	1.54178 Å
Crystal system	orthorhombic
Space group	*P* 2_1_ 2_1_ 2
Unit cell dimensions	a = 8.208(3) Å
	b = 29.476(9) Å
	c = 7.603(3) Å
Volume	1839(1) Å3
Z	4
Density (calculated)	2.662 Mg/m^3^
Absorption coefficient	35.798 mm^−1^
F(000)	1336
range for data collection	6 to 70°
Index ranges	9 ≤ h ≤ 9, 0 ≤ k ≤ 35, 0 ≤ l ≤ 9
Reflections collected	3806
Independent reflections	3486 [R(int) = 0.1424
Refinement method	Full-matrix least-squares on F^2^
Data/restraints/parameters	3486/00/176
Goodness-of-fit on F^2^	1.237
Final R indices [I > 2σ(I)]	R1 = 0.1314, wR2 = 0.3058
R indices (all data)	R1 = 0.1443, wR2 = 0.3343
Absolute structure parameter	0.01(7)
Extinction coefficient	0.00035(14)
Largest ΔF maximum/minimum	6.866 / −3.415 e.Å^−3^

## Supplementary Materials

CCDC 916392 contains the supplementary crystallographic data for this paper. These data can be obtained free of charge via https://www.ccdc.cam.ac.uk/structures/, or by mail to data_request@ccdc.cam.ac.uk, or by contacting The Cambridge Crystallographic Data Centre, 12 Union Road, Cambridge CB2 1EZ, UK; fax: +44(0)1223-336033; e-mail: deposit@ccdc.cam.ac.uk.
