# Supplementary material for: The Chiral 1:2 Adduct (S)S(S)C(−)589-Ethyl 2-Phenylbutyl Sulphide-Mercury (II) Chloride:(−)589[(S)S(S)C-Et(2-PhBu)S.(HgCl2)2]. Stereoselective Synthesis, Asymmetric Oxidation, Crystal and Molecular Structure and Circular Dichroism Spectra
Source: Molecules. 2020 Jul 27;25(15):3398. doi: 10.3390/molecules25153398 (PMC7435776; doi:10.3390/molecules25153398)
Supplement: Supplementary file 1 [file molecules-25-03398-s001.pdf]

SUPPORTING INFORMATION

The Chiral 1:2 Adduct (S)$_S$(S)$_C$(-)$_{589}$-Ethyl 2-Phenylbutyl Sulfide-Mercury (II) Chloride:(-)$_{589}$[(S)$_S$(S)$_C$-Et(2-PhBu)S.($HgCl_2$)]. Stereoselective synthesis, Asymmetric Oxidation, Crystaland Molecular Structure and Circular Dichroism Spectra

Paolo Biscarini.[1*] , Ivano Bilotti,[1] Francesco Ferranti,[1] Alessia Bacchi,[2] Giancarlo Pelizzi,[2]

Marian Mikołajczyk[3], Jozef Drabowicz,[3,4*]

1. Alma Mater Studiorum Bologna University, V.le Risorgimento 4 Bologna Italy; (formely "Toso Montanar" Department of Industrial Chemistry CHIMIND, Bologna Italy) e-mail: paolobiscarini@alice.it
2. Dipartimento di Chimica Generale ed Inorganica, Università di Parma, V.le delle Scienze, 43100 Parma; e-mail: chimic6@ipruniv.cce.unipr.it
3. Center of Molecular and Macromolecular Studies, Polish Academy of Sciences, Division of Organic Chemistry 90-363 Lodz, Sienkiewicza 112 , Poland; e-mail: draj@cbmm.lodz.pl
4. Jan Dlugosz University in Czestochowa, 42-200 Czestochowa, Armii Krajowej 13/15, Poland, e-mail: j.drabwicz@ajd.czest.pl

Table SI2. Bond lengths (Å) and angles (°) for (II) .

Hg1-Cl2	2.366(7)	Cl2-Hg1#2	3.333(7)
Hg1-S	2.397(7)	Cl4-Hg1#3	3.259(9)
Hg1-Cl5	2.767(5)	Cl4-Hg2#4	3.268(8)
Hg1-Cl1	2.827(8)	Cl5-Hg1#4	2.767(5)
Hg1-Cl4#1	3.259(9)	Cl5-Hg2#4	3.146(8)
Hg1-Cl2#2	3.333(7)	C1-C2	1.55(3)
Hg2-Cl3	2.276(8)	C2-C3	1.50(4)
Hg2-Cl4	2.300(7)	C2-C9	1.53(5)
Hg2-Cl1	3.046(6)	C3-C8	1.43(4)
Hg2-Cl5	3.146(8)	C3-C4	1.44(4)
Hg2-Cl2#3	3.162(8)	C4-C5	1.36(4)
Hg2-Cl4#4	3.268(8)	C5-C6	1.42(5)
S -C1	1.77(3)	C6-C7	1.38(7)
S -C11	1.86(2)	C7-C8	1.29(6)
Cl1-Hg1#2	2.827(8)	C9-C10	1.50(6)
Cl1-Hg2#2	3.046(6)	C11-C1	1.53(5)
Cl2-Hg2#1	3.162(8)		

Cl2-Hg1-S	154.9(3)	C11-S -Hg1	101.4(11)
Cl2-Hg1-Cl5	100.2(2)	Hg1-Cl1-Hg1#2	80.3(3)
S -Hg1-Cl5	97.9(2)	Hg1-Cl1-Hg2#2	145.9(2)
Cl2-Hg1-Cl1	90.4(2)	Hg1#2-Cl1-Hg2#2	89.16(5)
S -Hg1-Cl1	104.5(2)	Hg1-Cl1-Hg2	89.16(5)
Cl5-Hg1-Cl1	96.9(2)	Hg1#2-Cl1-Hg2	145.9(2)
Cl2-Hg1-Cl4#1	82.2(2)	Hg2#2-Cl1-Hg2	115.7(3)
S -Hg1-Cl4#1	76.5(2)	Hg1-Cl2-Hg2#1	96.6(2)
Cl5-Hg1-Cl4#1	104.7(2)	Hg1-Cl2-Hg1#2	77.4(2)
Cl1-Hg1-Cl4#1	158.1(2)	Hg2#1-Cl2-Hg1#2	120.6(2)
Cl2-Hg1-Cl2#2	90.1(2)	Hg2-Cl4-Hg1#3	95.4(3)
S -Hg1-Cl2#2	75.5(2)	Hg2-Cl4-Hg2#4	88.6(2)
Cl5-Hg1-Cl2#2	166.0(2)	Hg1#3-Cl4-Hg2#4	118.4(3)
Cl1-Hg1-Cl2#2	73.3(2)	Hg1-Cl5-Hg1#4	122.4(4)
Cl4#1-Hg1-Cl2#2	86.0(2)	Hg1-Cl5-Hg2#4	141.3(2)
Cl3-Hg2-Cl4	173.2(4)	Hg1#4-Cl5-Hg2#4	88.24(8)
Cl3-Hg2-Cl1	98.8(3)	Hg1-Cl5-Hg2	88.24(8)
Cl4-Hg2-Cl1	87.9(2)	Hg1#4-Cl5-Hg2	141.3(2)
Cl3-Hg2-Cl5	96.3(3)	Hg2#4-Cl5-Hg2	77.8(2)
Cl4-Hg2-Cl5	84.4(2)	C2-C1-S	114(2)
Cl1-Hg2-Cl5	85.1(2)	C3-C2-C9	114(3)
Cl3-Hg2-Cl2#3	92.4(3)	C3-C2-C1	114(3)
Cl4-Hg2-Cl2#3	85.4(3)	C9-C2-C1	109(3)
Cl1-Hg2-Cl2#3	106.1(2)	C8-C3-C4	115(3)
Cl5-Hg2-Cl2#3	164.6(2)	C8-C3-C2	122(3)
Cl3-Hg2-Cl4#4	91.3(3)	C4-C3-C2	123(3)
Cl4-Hg2-Cl4#4	82.6(3)	C5-C4-C3	125(3)
Cl1-Hg2-Cl4#4	154.7(2)	C4-C5-C6	116(3)
Cl5-Hg2-Cl4#4	70.7(2)	C7-C6-C5	119(3)
Cl2#3-Hg2-Cl4#4	96.5(2)	C8-C7-C6	125(4)
C1-S -C11	107(2)	C7-C8-C3	120(4)
C1-S -Hg1	103.6(9)	C10-C9-C2	118(4)
		C12-C11-S	111(2)

Symmetry transformations used to generate equivalent atoms:

#1 x,y,z-1   #2 -x,-y+1,z   #3 x,y,z+1

#4 -x+1,-y+1,z

checkcif.pdf

hgas1.cif
